# Comparison of Histologic Parameters and Predictive Gene Signatures in Clear Cell Renal Cell Carcinoma Response to Systemic Therapy

**DOI:** 10.1111/pin.70012

**Published:** 2025-05-27

**Authors:** Chisato Ohe, Takashi Yoshida, Mahul B. Amin, Steven C. Smith, Masanori Shiohara, Nozomi Tsujio, Masahiro Kato, Rena Uno, Toyonori Tsuzuki, Kenichi Kohashi

**Affiliations:** ^1^ Department of Pathology Graduate School of Medicine, Osaka Metropolitan University Osaka Japan; ^2^ Department of Urology and Andrology Kansai Medical University Osaka Japan; ^3^ Department of Urology Osaka Saiseikai‐Noe Hospital Osaka Japan; ^4^ Graduate School of Engineering Tottori University Tottori Japan; ^5^ Department of Pathology and Laboratory Medicine University of Tennessee Health Sciences Center Memphis Tennessee USA; ^6^ Department of Urology University of Southern California Los Angeles California USA; ^7^ Departments of Pathology and Urology, School of Medicine and VCU Massey Comprehensive Cancer Center Virginia Commonwealth University Richmond Virginia USA; ^8^ Department of Pathology Hyogo Cancer Center Hyogo Japan; ^9^ Department of Surgical Pathology Aichi Medical University Hospital Nagakute Aichi Japan

**Keywords:** architecture, clear cell renal cell carcinoma, eosinophilic phenotype, gene signature, histology, immunophenotype, prognosis, WHO/ISUP grade

## Abstract

Growing experience has correlated the histomorphological characteristics of clear cell renal cell carcinoma (ccRCC), ranging from cytoplasmic features to architectural patterns and tumor immune microenvironment, with clinical outcomes. However, further assessment is needed to determine which of these histologic parameters best correlate with outcomes of interest, especially response to tyrosine kinase inhibitors (TKIs) and immune checkpoint inhibitors (ICIs). Herein, we evaluated four histologic parameters: (i) World Health Organization (WHO)/International Society of Urological Pathology (ISUP) grade; (ii) clear and eosinophilic cytological phenotypes; (iii) immunophenotypes; and (iv) vascularity‐based architectural classification, using hematoxylin and eosin‐stained whole slide images for The Cancer Genome Atlas (TCGA) ccRCC cohort (*n* = 433). We then correlated these parameters with gene expression signatures associated with TKI and ICI response. Multivariate analysis found that the cytological phenotype and vascularity‐based architectural classification were independently associated with an angiogenesis‐related gene signature (both *p* < 0.05). Conversely, WHO/ISUP grade and immunophenotype were independently associated with effector T‐cell and immune checkpoint gene signatures (both *p* < 0.05). In conclusion, histologic parameters, including cytological features, architectural patterns, and tumor immune microenvironment, are associated with gene signatures related to therapy response, with different parameters informative for TKIs versus ICIs. These findings may help guide prospective validation studies.

## Graphical abstract

Histological parameters are associated with gene signatures predictive of TKI and ICI response. Cytological and architectural parameters correlate with angiogenesis‐related gene signature. WHO/ISUP grade and immunophenotype correlate with immune‐related gene signature.

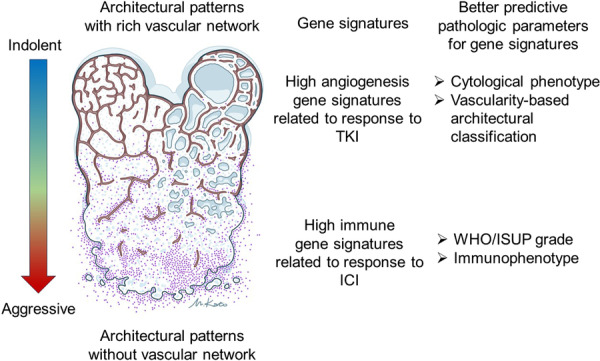

**Abbreviations**
ccRCC: clear cell renal cell carcinoma
HIF: hypoxia‐inducible factor
ICI: immune checkpoint inhibitor
ISUP: International Society of Urological Pathology
TAICs: tumor‐associated immune cells
TCGA: The Cancer Genome Atlas
TKI: tyrosine kinase inhibitor
VEGF: vascular endothelial growth factor
VHL: von Hippel−Lindau
WHO: World Health Organization
WSI: whole slide image

## 1 Introduction

Clear cell renal cell carcinoma (ccRCC) is characterized by its high vascularization, primarily due to the inactivation of the *von Hippel–Lindau* (*VHL*) tumor suppressor gene, leading to the overexpression of hypoxia‐inducible factor (HIF) and vascular endothelial growth factor (VEGF) [1]. Over the past two decades, treatment strategies for advanced ccRCC have evolved significantly with the widespread use of VEGF tyrosine kinase inhibitors (TKIs), immune checkpoint inhibitors (ICIs), and combinations of these approaches [2, 3, 4, 5].

Currently, treatment selection is guided by the International Metastatic RCC Database Consortium (IMDC) criteria based on clinical characteristics [6]. While the IMDC model effectively stratifies patients with ccRCCs undergoing TKI treatment, its applicability might be limited in the immuno‐oncology era [7, 8]. Recently, several transcriptomic signatures have been used to predict responses to TKIs and ICIs. These include angiogenesis‐related gene signatures for TKIs and immune‐related gene signatures for ICIs, such as those identified in the IMmotion 150 [9] or JAVELIN Renal 101 Immuno [10] studies. There is a growing need for simplified, optimal biomarkers that can accurately determine the best treatment options, reflect the underlying antitumor mechanisms at the genomic level, minimize costs, and improve patient outcomes [6, 11].

In efforts to identify histological features that could inform on key clinical phenotypes or even guide appropriate treatment selection in ccRCC, we, as well as others, have explored using cytoplasmic, architectural, and tumor immune microenvironment patterns to provide greater diagnostic, prognostic, and predictive precision for ccRCC patients [12, 13, 14, 15, 16, 17]. Such assessments could be used to complement the established World Health Organization/International Society of Urological Pathology (WHO/ISUP) grading system and internationally used cancer staging systems [18] to add value to management. To date, promising findings from our group have defined a family of histologic parameters, based on facile, routine hematoxylin and eosin (H&E) assessment alone, and associated them with salient clinical and therapeutic features: these parameters include a cytological phenotype (based on the degree of cytoplasmic eosinophilia) [13], a vascularity‐based architectural classification [16], and an immunophenotype based on the degree of and configuration of tumor inflammatory infiltrate [17]. Our studies have strongly associated these new histologic parameters individually with key gene signatures, protein expression, underlying genomic factors influencing TKIs and ICIs response, and even survival. However, before prospective testing of these histologic parameters with additional clinical cohorts, further research is required to determine the relationships between these higher‐order histologic parameters and assess which most strongly correlate with or predict outcomes or phenotypes of interest.

To that end, herein, we extend our prior studies to explore the association of each of these histologic parameters, alongside WHO/ISUP nucleolar grade, with each other, as well as with a set of recently published gene expression signatures that have been associated with responses to systemic therapy [9]. For this study, we use the well‐characterized and prognostically annotated Cancer Genome Atlas (KIRC‐TCGA) cohort with paired whole slide images (WSIs) and ribonucleic acid (RNA) sequencing data to explore these associations. The findings provide intriguing insights into how higher‐order histologic parameters, including our cytological phenotype, immunophenotype, and vascularity‐based architectural classification, correlate with key gene expression and prognostic outcomes, nominating histologic parameters for consideration for prospective testing in clinical cohorts.

## 2 Materials and Methods

### 2.1 Data Sources

From the initial cohort of 488 ccRCC cases reported in the KIRC‐TCGA database [19], 55 cases were excluded due to the unavailability or insufficiency of WSIs and RNA sequencing data. Ultimately, 433 ccRCC cases were included in our analysis. For each case, a representative H&E‐stained WSI was retrieved from the Digital Slide Archive [20], and relevant clinical variables, such as gender, pathological stage, and survival information, were extracted from previously published data [19]. This same cohort was also used in our previous study [21].

### 2.2 Histopathological Evaluation

**Figure 1:**
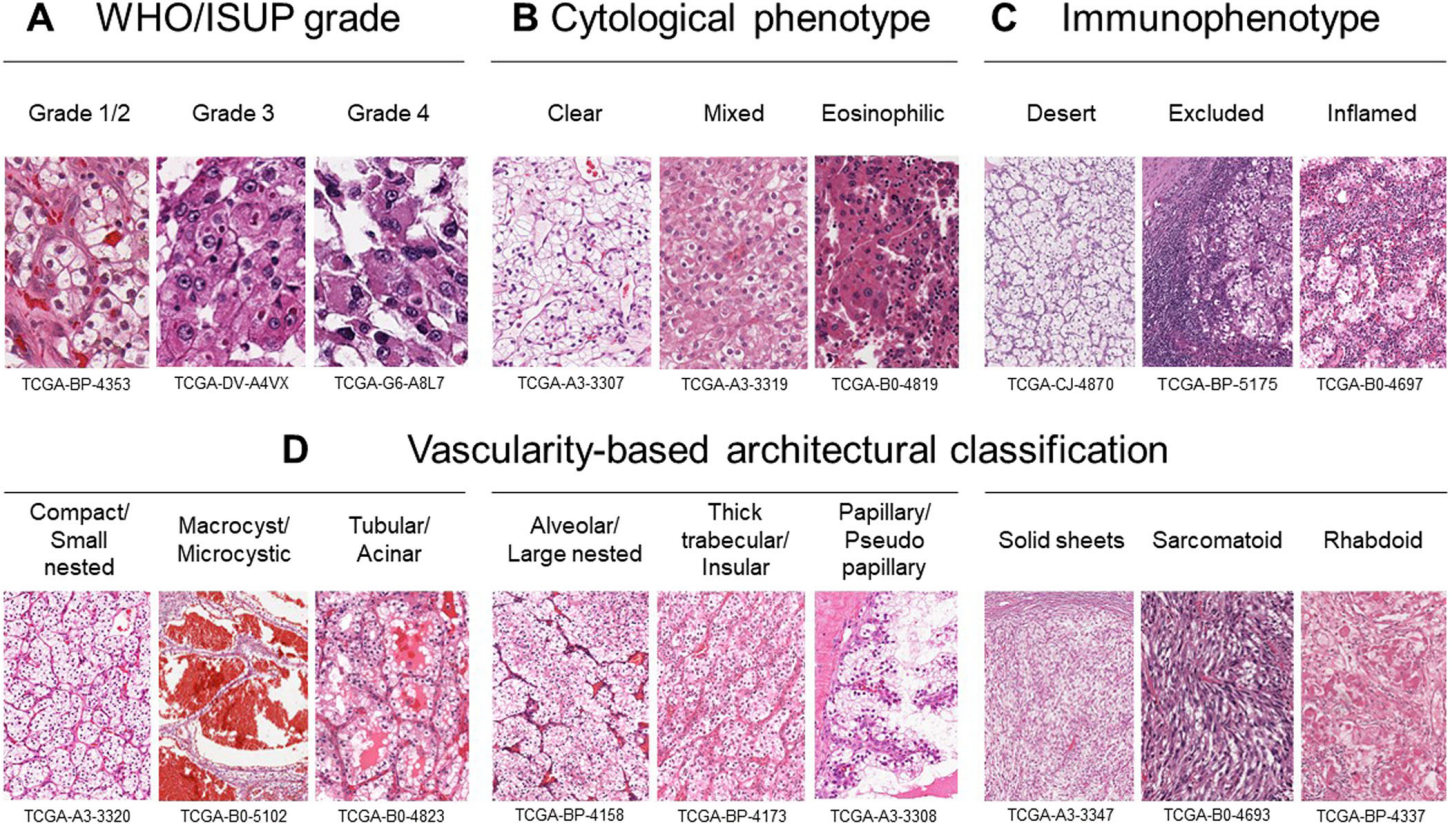
Representative hematoxylin and eosin images illustrating pathological parameters. The World Health Organization/International Society of Urological Pathology grade (A), cytological phenotypes (B), immunophenotypes (C), and vascularity‐based architectural classification (D) [13, 16, 17].

Four histologic parameters were assessed in the study: (i) WHO/ISUP nucleolar grade [22] (Figure 1A), (ii) cytological phenotypes, categorized as clear, mixed, or eosinophilic [13, 14] (Figure 1B), (iii) immunophenotypes, classified as desert, excluded, or inflamed [17] (Figure 1C), and (iv) a vascularity‐based architectural classification [16] (Figure 1D). The vascularity‐based architectural classification was divided into three categories: category 1, characterized by an enriched vascular network, encompassing compact/small nested, macrocystic/microcystic, and tubular/acinar patterns; category 2, characterized by a widely spaced vascular network, including alveolar/large nested, thick trabecular/insular, and papillary/pseudopapillary patterns; and category 3 characterized by scattered vascularity without a vascular network, including solid sheets, sarcomatoid, and rhabdoid patterns. All H&E images were evaluated by a urologic pathologist (C.O.) who was blinded to the clinical variables.

Examples for determining cytological phenotype, immunophenotype, and vascularity‐based architectural classification are provided in Figure S1. The evaluation process began with a low‐power scan of the entire slide to identify the area with the highest WHO/ISUP grade. Within this high‐grade area, the cytological phenotype and architectural classification were determined, provided they appeared in at least one high‐power field, following the criteria established for WHO/ISUP grading [22]. In contrast, for assessment of the immunophenotype (an H&E‐based assessment of immune cells performed across the entire section at lower power), the presence and configuration of tumor‐associated immune cells (TAICs) was evaluated, including both mononuclear cells and granulocytes. The classification of TAICs was based on their location within the representative WSI, independent of their density, and categorized into three types: desert (absence of TAICs), excluded (TAICs restricted to the peritumoral region), and inflamed (intratumoral TAICs) as detailed previously [17].

### 2.3 Gene Expression Analysis

To evaluate the association between four histologic parameters and gene signatures related to therapeutic response, relevant genes, including those from the IMmotion 150 gene panel [9], which encompasses angiogenesis, immune and antigen presentation, and myeloid inflammation pathways, from the TCGA RNA sequencing data, were extracted, as previously described [14, 16, 17, 21]. Four gene signatures were defined [16]: angiogenesis (*VEGFA, KDR, ESM1, PECAM1, FLT1, ANGPTL4*, and *CD34*); effector T cell (*CD8A, INFG, GZMA, GZMB, PRF1*, and *EOMES*); immune checkpoint (*CD274 (PD‐L1), CTLA4*, and *TIGIT*); and myeloid (*CXCL1, CXCL2, CXCL3, IL6*, and *PTGS2*). Gene signature scores were calculated by *Z*‐score normalizing (across all tumors) the expression value of each of these genes, and then averaging normalized scores to create a signature score for each of these signatures for each patient.

### 2.4 Statistical Analysis

Statistical analyses were performed using EZR version 1.54 (Saitama Medical Center, Jichi, Japan) [23]. Continuous data are presented as median values and interquartile ranges (IQRs). The Kruskal−Wallis test was applied to assess the statistical significance of differences among the three groups for nonparametric variables. Multiple linear regression analysis was performed to identify the influence of histologic parameters on gene signatures. Overall survival (OS), defined as the time from surgery to death from any cause, was analyzed using the Kaplan–Meier method with log‐rank tests, as well as the Cox proportional hazards model. Harrell's concordance index (C‐index) was used to evaluate the predictive accuracy of the Cox models. Statistical significance was set at a *p*‐value of < 0.05.

## 3 Results

### 3.1 Clinicopathological Characteristics and Histologic Parameters

Table 1 presents the clinicopathological characteristics and outcomes of the 433 patients included in the study. Among these patients, 170 (39.2%) had advanced pathological prognostic factors, with TNM stage III or IV, and 218 (50.4%) had WHO/ISUP grade 3 or 4 tumors. During a median follow‐up period of 1200 days (IQR: 562−1913), 142 patients (32.8%) with ccRCC died.

**Table 1:** Clinicopathological characteristics of 433 cases with ccRCCs in the KIRC‐TCGA cohort.

Variables	
Gender, *n* (%)	
Female	159 (36.7)
Male	274 (63.3)
TNM stage, *n* (%)	
I	217 (50.1)
II	46 (10.6)
III	107 (24.7)
IV	63 (14.5)
WHO/ISUP grade, *n* (%)	
1	29 (6.7)
2	186 (43.0)
3	154 (35.6)
4	64 (14.8)
Cytological phenotype, *n* (%)	
Clear	162 (37.4)
Mixed	229 (52.9)
Eosinophilic	42 (9.7)
Immunophenotype, *n* (%)	
Desert	189 (43.6)
Excluded	90 (20.8)
Inflamed	154 (35.6)
Vascularity‐based architectural classification, *n* (%)	
Category 1	182 (42.0)
Category 2	183 (42.3)
Category 3	68 (15.7)
Overall mortality, *n* (%)	142 (32.8)

Abbreviations: ccRCC, clear cell renal cell carcinoma; ISUP, International Society of Urological Pathology; WHO, World Health Organization.

With respect to the histologic parameters studied, the distribution of cytological phenotypes was as follows: 162 (37.4%) patients exhibited the clear phenotype, 229 (52.9%) exhibited the mixed phenotype, and 42 (9.7%) presented with the eosinophilic phenotype. Regarding immunophenotypes, 189 (43.6%) patients were classified as having the desert type, 90 (20.8%) patients the excluded type, and 154 (35.6%) patients the inflamed type. Vascularity‐based architectural classifications were distributed as follows: 182 (42.0%) patients were classified into category 1, 183 (42.3%) patients into category 2, and 68 (15.7%) patients into category 3.

### 3.2 Association Histologic Parameters and Gene Expression Signatures Associated With Response to TKI and ICI Therapy

**Figure 2:**
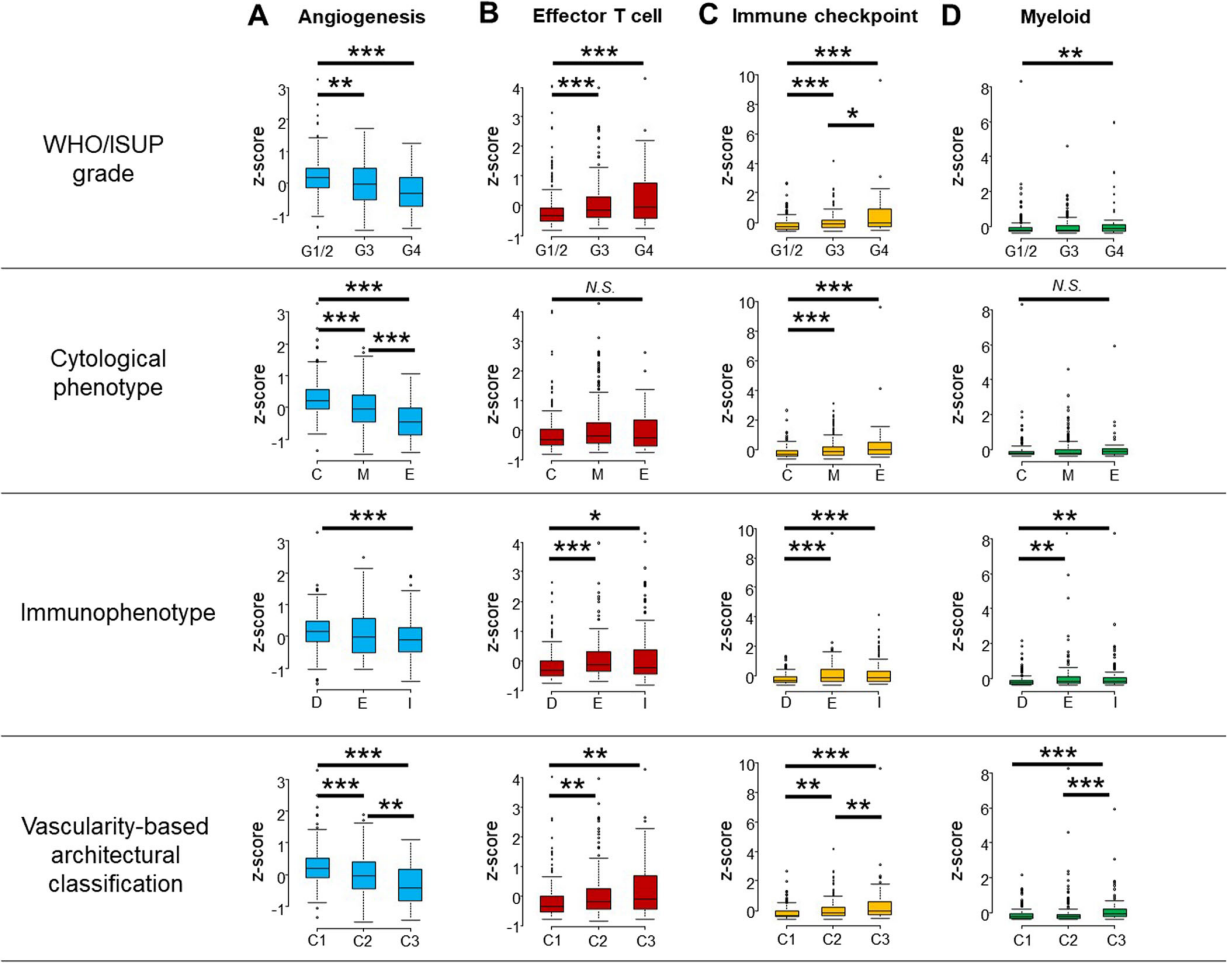
Association of histologic parameters with gene expression signatures (mean *Z*‐score). Angiogenesis‐related gene signature (A), effector T cell gene signature (B), immune checkpoint gene signature (C), and myeloid gene signature (D). Statistical significance was assessed using the Kruskal−Wallis test (NS = not statistically significant; *p < 0.05; **p < 0.01; ***p < 0.001). C, category; C, clear; D, desert; E, eosinophilic; E, excluded; G, grade; I, inflamed; and M, mixed.

Figure 2 illustrates the associations between histologic parameters and gene signatures. For angiogenesis‐related gene signatures, significant differences were observed across all four histologic parameters (*p* < 0.001). Angiogenesis gene scores were significantly higher in cases with WHO/ISUP grade 1/2, the clear phenotype, and vascularity‐based category 1 compared to those with WHO/ISUP grade 3/4, mixed and eosinophilic phenotypes, and vascularity‐based categories 2 and 3. Additionally, significant differences were observed between the mixed and eosinophilic phenotypes, as well as vascularity‐based categories 2 and 3 (*p* < 0.001 and *p* < 0.01, respectively).

In contrast, immune checkpoint gene signatures exhibited a reverse trend to angiogenesis‐related gene signatures, with significant differences across all four pathological factors (*p* < 0.001). Immune checkpoint gene scores were elevated cases with WHO/ISUP grade 4, the eosinophilic phenotype, the inflamed phenotype, and vascularity‐based category 3 compared to those with WHO/ISUP grade 1/2 and 3, clear and mixed phenotypes, desert and excluded immunophenotypes, and vascularity‐based categories 1 and 2. Significant differences were also observed between WHO/ISUP grade 3/4 and vascularity‐based categories 2 and 3 (*p* < 0.05 and *p* < 0.01, respectively).

Regarding effector T cell and myeloid gene signatures, significant differences were observed across the WHO/ISUP grades, immunophenotypes, and vascularity‐based classifications (*p* < 0.05). However, no significant differences were observed in relation to the cytological phenotypes.

### 3.3 Multivariate Analysis of Histologic Parameters and Gene Signatures

The results of multiple linear regression analyses are shown in Table 2. The multivariate analysis found that the cytological phenotype and vascularity‐based architectural classification were both independently associated with angiogenesis‐related gene signatures (both *p* < 0.05). In contrast, WHO/ISUP grade (grade 4 vs. 1/2 for both) and immunophenotype (inflamed vs. desert for effector T‐cell; excluded vs. desert for immune checkpoint) were independently associated with effector T‐cell and immune checkpoint gene signatures (both *p* < 0.05). Immunophenotype (excluded vs. desert) and vascularity‐based architectural classification (category 3 vs. 1) were independently associated with myeloid gene signature (both *p* < 0.05).

**Table 2:** Multivariate analysis of associations between pathological factors and gene signatures.

Variables	Angiogenesis			Effector T cell			Immune checkpoint			Myeloid		
	Coefficient (95％ CI)	SE	*p* value	Coefficient (95％ CI)	SE	*p* value	Coefficient (95％ CI)	SE	*p* value	Coefficient (95％ CI)	SE	*p* value
WHO/ISUP grade												
Grade 3 versus 1/2	0.004 (−0.15 to 0.16)	0.078	0.954	0.141 (−0.05 to 0.34)	0.099	0.156	−0.004 (−0.19 to 0.18)	0.096	0.967	−0.008 (−0.18 to 0.17)	0.089	0.929
Grade 4 versus 1/2	−0.090 (−0.31 to 0.13)	0.112	0.423	0.289 (0.01 to 0.57)	0.143	**0.043**	0.281 (0.01 to 0.55)	0.138	**0.043**	0.008 (−0.24 to 0.26)	0.128	0.952
Cytological phenotype												
Mixed versus clear	−0.221 (−0.36 to −0.08)	0.072	**0.002**	−0.024 (−0.20 to 0.16)	0.092	0.798	0.091 (−0.08 to 0.27)	0.089	0.304	−0.058 (−0.22 to 0.10)	0.082	0.484
Eosinophilic versus clear	−0.453 (−0.71 to −0.19)	0.132	**< 0.001**	−0.320 (−0.65 to 0.01)	0.168	0.058	0.225 (−0.09 to 0.54)	0.163	0.167	−0.050 (−0.35 to 0.25)	0.151	0.741
Immunophenotype												
Excluded versus desert	0.054 (−0.12 to −0.23)	0.087	0.531	0.197 (−0.02 to 0.41)	0.110	0.074	0.314 (0.10 to 0.52)	0.107	**0.003**	0.214 (0.02 to 0.41)	0.099	**0.032**
Inflamed versus desert	−0.006 (−0.16 to 0.15)	0.077	0.940	0.206 (0.01 to 0.40)	0.098	**0.036**	0.176 (−0.01 to 0.36)	0.095	0.063	0.094 (−0.08 to 0.27)	0.088	0.286
Vascularity‐based architectural classification												
Category 2 versus 1	−0.167 (−0.32 to −0.02)	0.076	**0.028**	0.087 (−0.10 to 0.28)	0.096	0.364	0.051 (−0.13 to 0.23)	0.093	0.582	0.054 (−0.12 to 0.22)	0.086	0.532
Category 3 versus 1	−0.284 (−0.52 to −0.05)	0.121	**0.019**	0.272 (−0.03 to 0.57)	0.153	0.076	0.240 (−0.05 to 0.53)	0.149	0.106	0.294 (0.02 to 0.57)	0.138	**0.034**

*Note:* Bold value indicates *p* < 0.05.

Abbreviations: CI, confidence interval; SE, standard error.

### 3.4 Prognostic Significance of the Four Pathologic Parameters

**Figure 3:**
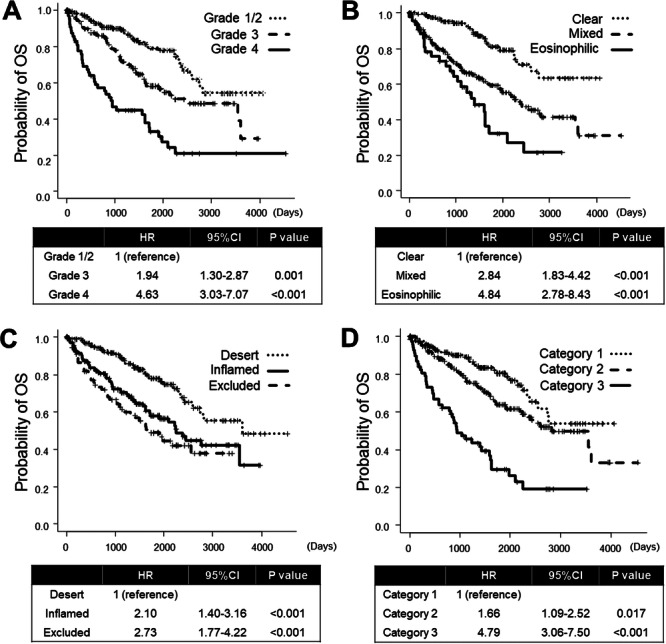
Comparison of prognostic significance among histologic parameters. Kaplan−Meier curves for the World Health Organization/International Society of Urological Pathology grade (A), cytological phenotypes (B), immunophenotypes (C), and vascularity‐based architectural classification (D). CI, confidence interval; HR, hazard ratio; OS, overall survival.

Kaplan−Meier survival analysis revealed a 5‐year OS rate of 58.1% for patients with WHO/ISUP grade 3 tumors (hazard ratio [HR]: 1.94; *p* = 0.001) and 32.9% (HR: 4.63; *p *< 0.001) for those with grade 4 tumors, compared to 79.2% for those with grade 1 tumors (Figure 3A). Similarly, the 5‐year OS rate was 59.2% (HR: 2.84; *p* < 0.001) for patients with the mixed phenotype and 32.1% (HR: 4.84; *p *< 0.001) for those with the eosinophilic phenotype compared to 80.9% for those with the clear phenotype (Figure 3B). For immunophenotypes, the 5‐year OS rate was 57.9% (HR: 2.10; *p* < 0.001) for the inflamed type and 48.8% (HR: 2.37; *p *< 0.001) for the excluded type, compared to 77.9% for the desert type (Figure 3C). Regarding the vascularity‐based architectural classification, the 5‐year OS rate was 63.7% (HR: 1.66; *p* = 0.017) for category 2 and 29.4% (HR: 4.79; *p *< 0.001) for category 3, compared to 80.5% for category 1 (Figure 3D). Among the variables examined, the WHO/ISUP grade demonstrated the highest C‐index for predicting OS, outperforming the vascularity‐based architectural classification, cytological phenotypes, and immunophenotypes (0.672 vs. 0.658, 0.648, and 0.634, respectively). On multivariate analysis, the cytological phenotype (mixed vs. clear, HR: 1.910; *p* < 0.010) and vascularity‐based architectural classification (category 3 vs. 1, HR: 1.912; *p* < 0.040) were both identified as an independent prognostic factor for OS as well as WHO/ISUP grade (grade 4 vs. 1/2, HR: 2.120; *p *< 0.008) (Table 3).

**Table 3:** Cox regression analysis of prognostic factors for predicting overall survival in ccRCCs of the TCGA cohort.

Variables	Univariate		Multivariate		
	*p* value	*c*‐index	HR	95% CI	*p* value
WHO/ISUP grade		0.672			
Grade 1/2	—		1 (ref.)		
Grade 3	< 0.001		1.237	0.78−1.97	0.370
Grade 4	< 0.001		2.120	1.22−3.69	0.008
Cytological phenotype		0.648			
Clear	—		1 (ref.)		
Mixed	< 0.001		1.910	1.17−3.13	0.010
Eosinophilic	< 0.001		1.917	0.98−3.76	0.058
Immunophenotype		0.634			
Desert	—		1 (ref.)		
Excluded	< 0.001		1.564	0.96−2.54	0.071
Inflamed	< 0.001		1.205	0.76−1.91	0.425
Vascularity‐based architectural classification		0.658			
Category 1	—		1 (ref.)		
Category 2	< 0.001		0.051	0.65−1.70	0.840
Category 3	< 0.001		1.912	1.03−3.55	0.040
*c*‐index	—		0.735		

Abbreviations: ccRCC, clear cell renal cell carcinoma; CI, confidence interval; HR, hazard ratio; ISUP, International Society of Urological Pathology; WHO, World Health Organization.

## 4 Discussion

In this study, using the KIRC‐TCGA cohort with RNA sequencing data, it was found that among the histologic parameters studied for associations with signatures of therapy response, the cytological phenotype and vascularity‐based architectural classification were independently associated with angiogenesis‐related gene signature. Conversely, WHO/ISUP grade and immunophenotype were independently associated with effector T‐cell and immune checkpoint gene signatures. While the WHO/ISUP grade remains the most robust predictor of OS, classifying cytoplasmic features, architectural patterns, and the histologically determined tumor immune microenvironment, alongside the WHO/ISUP grade, could aid in the selection of an appropriate systemic therapy for ccRCC.

Distinct gene expression signatures associated with angiogenesis and immune infiltration have been shown to correlate with therapeutic responses [9, 10, 24, 25]. In this study, angiogenesis‐related, effector T cell, immune checkpoint‐related, and myeloid gene signatures were used, validating them against the gene panels from the IMmotion 150 trial, a randomized phase 2 study comparing atezolizumab (anti‐PD‐L1) alone or in combination with bevacizumab (anti‐VEGF) versus sunitinib [9]. This clinical trial demonstrated that patients in the angiogenesis‐high subgroup were more likely to respond to TKIs, while those in the T‐effector‐high group responded better to the combination of ICIs and TKIs. Our study revealed that among the histologic parameters studied, the cytological phenotype and vascularity‐based architectural classification were associated with angiogenesis‐related gene signatures, while WHO/ISUP grade and immunophenotype were associated with immune‐related gene signatures. Notably, this study is the first to compare pathologic parameters that reflect gene signatures related to TKI and ICI responsiveness with conventional WHO/ISUP grades.

The clinical significance of the WHO/ISUP grading system for ccRCC has been thoroughly validated. Notably, ccRCC cases with prominent and eosinophilic nucleoli (WHO/ISUP grade 3) are associated with poorer outcomes compared to those with inconspicuous or absent nucleoli. In contrast, WHO/ISUP grade 4 is characterized by nuclear anaplasia, which includes features such as tumor giant cells, sarcomatoid differentiation, and/or rhabdoid morphology [22]. In cases of ccRCC with sarcomatoid and rhabdoid differentiation—tumors known for their high aggressiveness—the combination of ICIs has shown potential for improved responses and outcomes, likely due to immune‐inflamed phenotype and elevated PD‐1 and PD‐L1 expression [26, 27]. Consistent with previous studies, our findings demonstrated significant enrichment of effector T cell and immune checkpoint gene signatures in WHO/ISUP grade 4 tumors. However, these forms of ccRCC are rare, with sarcomatoid features present in only 4%−5% of all RCCs and rhabdoid features reported in approximately 15% of sarcomatoid RCCs [26]. Therefore, the identification of additional mechanism‐driven biomarkers is crucial to guide therapeutic decisions better.

PD‐L1 expression, increased CD8+ tumor‐infiltrating lymphocyte (TIL) density, and a high mutational load are established biomarkers for predicting response to ICIs in solid tumors [28, 29]. Among the various parameters considered essential for selecting first‐line treatment in metastatic ccRCC, international experts have highlighted the importance of PD‐L1 status alongside the IMDC risk group [30]. However, the assessment of PD‐L1 status in routine clinical practice remains suboptimal due to inconsistencies across clinical trials, including variations in diagnostic antibodies (clone 28‐8, 22C3, SP142, or SP263), immunohistochemical platforms/protocols (Dako vs. Ventana), cell types (tumor cells or immune cells), and thresholds [31]. While CD8+ TIL distribution is commonly evaluated, our previous study demonstrated that assessment of TAICs by routine H&E microscopy, accounting for both the location/configuration and extent of TAICs, correlates with the immunohistochemical expression of CD8 and PD‐L1, as well as with gene expression signatures associated with cancer immunity [18]. Recent single‐cell transcriptomic analyses have provided novel insights into the relationship between ICI therapeutic response and immune cells, including various lymphoid cells, myeloid cells, and macrophages, in addition to CD8+ TILs [32, 33, 34]. Consistent with these findings, a comprehensive assessment of TAICs through our immunophenotype histologic parameter offers a potentially effective and facile methodology for evaluating the immune cell milieu of ccRCCs using routine sections.

Recent genomic advances have revealed that intratumoral heterogeneity in ccRCC is driven by multiple genetically distinct subclones. The typical histological features of ccRCC include neoplastic cells with clear cytoplasm and a delicate vascular network, which are activated by HIF following *VHL* gene inactivation [1]. An evolutionary study by the TRAcking Cancer Evolution through Therapy consortium identified frequent driver mutations in　*SETD2, BAP1, KDM5C, MTOR, PIK3CA, PTEN, p53*, and *KDM6A*, often occurring at the subclone level [35]. Integrating molecular and histologic perspectives, Kapur et al. proposed an architectural evolutionary model, identifying nine architectural patterns of ccRCC and associated vascular framework highlighted by CD31 immunohistochemical labeling, which has significant prognostic and therapeutic implications [36]. Their findings indicate that tumor architecture can predict future behavior: indolent behavior is associated with microcystic, tubular/acinar, bleeding follicles, and compact small nest patterns, while aggressive behavior is related to alveolar, papillary/pseudopapillary, thick trabecular/insular, and solid sheet patterns. Furthermore, a multiscale framework incorporating 33 parameters across three axes—architecture, cytology, and microenvironment—has been developed [15]. However, the application of these histological parameters in routine clinical practice has not been thoroughly investigated.

**Figure 4:**
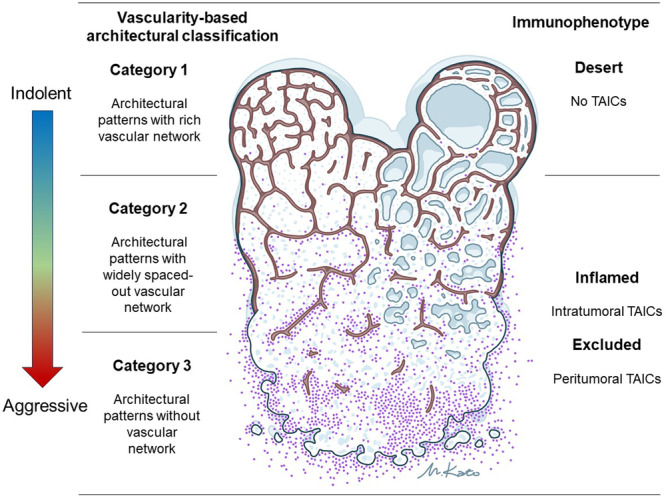
Schematic representation of the associations among neoplastic cells, vascularity‐based architectural classification, and immunophenotypes in clear cell renal cell carcinoma. Neoplastic cells are depicted in blue, the intervening vascular network in brown, and tumor‐associated immune cells in purple.

To simplify this model, our group developed a vascularity‐based architectural classification system, categorizing tumors into three groups based on vascular network score and the presence of sarcomatoid/rhabdoid features. Category 1 includes compact/small nested, macrocystic/microcystic, and tubular/acinar patterns with a rich vascular network. Category 2 comprises alveolar/large nested, thick trabecular/insular, and papillary/pseudopapillary patterns characterized by a widely spaced vascular network. Category 3 comprises solid sheet, sarcomatoid, and rhabdoid patterns, which exhibit scattered vasculature without a vascular network [16]. These categories were positively correlated with angiogenesis‐related gene signatures and inversely correlated with immune‐related gene signatures, suggesting an inverse relationship between vascularity and inflammatory status in ccRCC. To illustrate these findings, a schema summarizing the associations among neoplastic cells, vasculature, and inflammation in ccRCC was developed (Figure 4).

Recently, Subramanian et al. have shown that angio‐immune subtypes of tumor microenvironment based on transcriptomic analysis can predict therapeutic outcomes of ICIs [37]. They revealed a clear inverse relationship between angiogenesis and anti‐tumor immunity in various solid tumors, including RCC. Remarkably, patients displaying tumor microenvironment with low angiogenesis and high immune activity showed the most significant responses to ICI therapy. This finding suggests that vascularity‐based architectural category 3 (architectural pattern without vascular network with immune cell infiltration) is the most effective for ICI treatment response. Furthermore, we previously reported that HIF‐2α protein and messenger RNA expression were closely associated with vascularity‐based architectural category 1 (architectural pattern with rich vascular network without immune cell infiltration) [21]. The HIF‐2α inhibitor has recently been approved by the Food and Drug Administration for treating *VHL*‐mutated cancers [38], with potential future applications in advanced ccRCC [39]. Therefore, this vascularity‐based architectural classification might serve as a valuable tool for predicting responses to novel treatment strategies.

Our study has certain limitations. First, the evaluation was limited to the highest‐grade area in a single representative H&E‐stained WSI, without considering any other histology not represented in this sample. Second, given the limitation to a single WSI and assessment of the highest‐grade areas, the analysis did not account for intratumoral heterogeneity, a known factor for influencing tumor behavior [40]. Future research will use digital pathology to quantify histological parameters and correlate intratumoral heterogeneity with gene signatures, prognosis, and treatment response. Third, foreseeably, the tumor content available for assessment of the H&E‐stained WSI slides might not fully represent that analyzed in the RNA sequencing data due to the design of the TCGA experiment and its database. Fourth, our current immune checkpoint gene signature extracted from the IMmotion 150 gene panel, does not permit assessment of its correlation with ICI response in this study. Future studies will incorporate additional immune checkpoint genes to enable such analysis. Despite these limitations, remarkably, the clinical relevance of cytological, architectural, and tumor immune microenvironment histologic parameters of ccRCC was nonetheless demonstrated [13, 16, 17]. Although our study evaluated a limited set of gene signatures, further research is warranted. Future studies should focus on spatial gene signature analysis [41, 42] to better correlate histologic parameters with novel potential therapeutic targets.

In conclusion, this study provides further support for the premise that the integration of histologic parameters, including cytoplasmic features, architectural patterns, and histologic assessment of the tumor immune microenvironment, alongside established parameters such as the WHO/ISUP grade, has the potential to add predictive and prognostic value to the diagnosis of ccRCC. Most remarkable, in a time of comprehensive genomics and multiplexed biomarkers, these histologic parameters were sourced solely from the assessment of routine H&E‐stained slides, supporting the ongoing potential for refinement of routine surgical pathology assessment for personalized management. Intriguingly, multivariate analysis associated particular histologic parameters independently with gene expression signatures of each of the two overall categories of systemic therapy for ccRCC (TKIs and ICIs), nominating specific histologic parameters as biomarkers for prospective testing against response for specific therapies. Based on these findings, we continue to explore these histologic parameters as an emerging opportunity in the surgical pathology of kidney tumors.

## Author Contributions

**Chisato Ohe, Takashi Yoshida,** and **Mahul B. Amin:** conceptualization. **Chisato Ohe** and **Masanori Shiohara:** data curation and investigation. **Takashi Yoshida** and **Masanori Shiohara:** formal analysis. **Nozomi Tsujio, Masahiro Kato,** and **Rena Uno:** visualization and resources. **Chisato Ohe:** funding acquisition. **Chisato Ohe, Steven C. Smith, Masanori Shiohara,** and **Rena Uno:** methodology. **Chisato Ohe:** writing – original draft. **All authors:** writing – critical review and editing and final approval of the manuscript.

## Ethics Statement

Not applicable, as this study used only publicly available data from the Cancer Genome Atlas.

## Conflicts of Interest

Chisato Ohe has received honoraria for speaking engagements from Merck Sharp & Dohme. Kenichi Kohashi and Toyonori Tsuzuki are Editorial Board members of Pathology International and co‐authors of this article. To minimize bias, they were excluded from all editorial decision‐making related to the acceptance of this article for publication. The other authors declare no conflicts of interest.

## Supporting information


**Supporting Figure 1.** Examples of how to determine the pathological parameters.

## Data Availability

The data are available upon reasonable request by contacting the corresponding author.
