# Supplementary material for: Comparison of Histologic Parameters and Predictive Gene Signatures in Clear Cell Renal Cell Carcinoma Response to Systemic Therapy
Source: Pathol Int. 2025 May 27;75(6):267–77. doi: 10.1111/pin.70012 (PMC12184299; doi:10.1111/pin.70012)
Supplement: Supplementary file 1 — Supporting Figure 1. Examples of how to determine the pathological parameters. [file PIN-75-267-s001.pptx]

## Slide 1
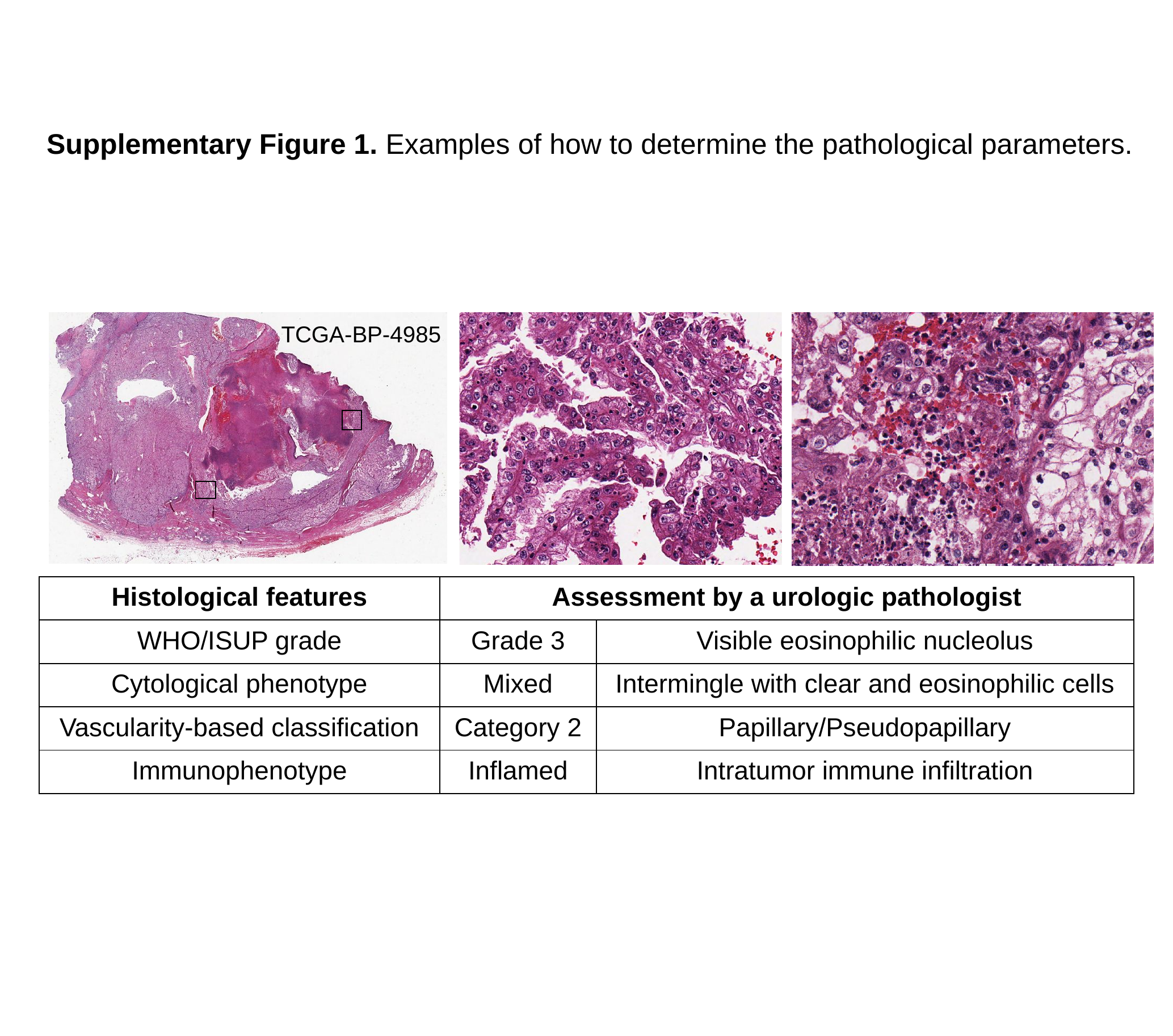

Supplementary Figure 1. Examples of how to determine the pathological parameters.
TCGA-BP-4985
| Histological features | Assessment by a urologic pathologist | |
| --- | --- | --- |
| WHO/ISUP grade | Grade 3 | Visible eosinophilic nucleolus |
| Cytological phenotype | Mixed | Intermingle with clear and eosinophilic cells |
| Vascularity-based classification | Category 2 | Papillary/Pseudopapillary |
| Immunophenotype | Inflamed | Intratumor immune infiltration |
